# Cost-effectiveness of enzyme replacement therapy for Fabry disease

**DOI:** 10.1186/1750-1172-8-29

**Published:** 2013-02-19

**Authors:** Saskia M Rombach, Carla EM Hollak, Gabor E Linthorst, Marcel GW Dijkgraaf

**Affiliations:** 1 Department of Internal Medicine, Division of Endocrinology and Metabolism, Academic Medical Centre, PO Box 22660, 1100, Amsterdam, DD, The Netherlands; 2 Clinical Research Unit. Academic Medical Centre, PO Box 22660, 1100, Amsterdam, DD, The Netherlands

## Abstract

**Background:**

The cost-effectiveness of enzyme replacement therapy (ERT) compared to standard medical care was evaluated in the Dutch cohort of patients with Fabry disease.

**Methods:**

Cost-effectiveness analysis was performed using a life-time state-transition model. Transition probabilities, effectiveness data and costs were derived from retrospective data and prospective follow-up of the Dutch study cohort consisting of males and females aged 5–78 years. Intervention with ERT (either agalsidase alfa or agalsidase beta) was compared to the standard medical care. The main outcome measures were years without end organ damage (renal, cardiac en cerebrovascular complications), quality adjusted life years (QALYs), and costs.

**Results:**

Over a 70 year lifetime, an untreated Fabry patient will generate 55.0 years free of end-organ damage (53.5 years in males, 56.9 years in females) and 48.6 QALYs (47.8 in males, 49.7 in females). Starting ERT in a symptomatic patient increases the number of years free of end-organ damage by 1.5 year (1.6 in males, 1.3 in females), while the number of QALYs gained increases by a similar amount (1.7 in males, 1.4 in females). The costs of ERT starting in the symptomatic stage are between €9 - €10 million (£ 7.9 - £ 8.8 million, $13.0- $14.5 million) during a patient’s lifetime. Consequently, the extra costs per additional year free of end-organ damage and the extra costs per additional QALY range from €5.5 - €7.5 million (£ 4.8 – £ 6.6 million, $ 8.0 – $ 10.8 million), undiscounted.

**Conclusions:**

In symptomatic patients with Fabry disease, ERT has limited effect on quality of life and progression to end organ damage. The pharmaco-economic evaluation shows that this modest effectiveness drives the costs per QALY and the costs per year free of end-organ damage to millions of euros. Differentiation of patients who may benefit from ERT should be improved to enhance cost-effectiveness.

## Background

Fabry disease (McKusick 301500) is a rare X-linked inherited multisystem lysosomal storage disorder, with an estimated birth prevalence around 1:40,000 [1,2]. Due to a deficiency of alfa-galactosidase A, globotriaosylceramide is stored in various cell types [3]. In hemizygous males, the signs or symptoms include acroparesthesia, inability to sweat, proteinuria, cardiac hypertrophy and cerebral ischemic lesions. Heterozygous females and atypical cases show a more variable and attenuated disease course [4,5]. The shortened life expectancy and the morbidity are clearly related to the degree of end-organ damage: progressive renal failure, heart failure, and stroke [4,6,7].

In 2002, the EMA approved two recombinant enzymes: agalsidase alfa (Shire HGT, Boston MA, USA) and agalsidase beta (Genzyme Inc, Boston MA, USA). Both received an orphan drug status and have been authorised under “Exceptional Circumstances”, which implicates a continuing lack of comprehensive clinical trial data due to the rarity of the disease. With the increasing number of orphan drugs and their extreme costs [8], there is need for more transparency of pricing and reimbursement of orphan drugs, including cost-effectiveness analyses [9]. So far, in the UK, effectiveness and cost-effectiveness of enzyme replacement therapies for Fabry disease and mucopolysaccharidosis type 1 have been investigated [10]. Under the assumption that life expectancy and morbidity were completely resolved after treatment, the investigators reported an optimistic incremental cost per additional QALY of 252,000 UK pounds. An even lower estimate of the incremental costs per QALY gained with ERT (about US $300,000) has been calculated by others [11]. Still, giving current market pricing, effectiveness evidence, and efficiency standards for health care, ERT is unlikely to be cost-effective. From a rights-based approach though, it may be argued that individuals are entitled to a decent minimum of health care, including treatment for rare diseases [12].

The Dutch government, by its healthcare insurance board requires the performance of health-economic analyses from a societal perspective for all orphan drugs. The Academic Medical Center in Amsterdam (AMC) was appointed as the coordinating center for the appraisal of enzyme replacement therapy for patients with Fabry disease. A life-time Markov-model was constructed to include the longer term consequences of treatment. The costs per year without end-organ damage and the costs per quality adjusted life-year (QALY) constituted the primary outcome measures.

## Methods

### Model structure

A life-time Markov state-transition model of the course of Fabry disease was used to evaluate the costs and effects of ERT against standard medical care. The model comprised eleven disease states including death (Additional file 1 Model):

○ *No symptoms* (no left ventricular hypertrophy, kidney disease, white matter lesions or complications)

○ *Acroparesthesia* (neuropathic pain in the extremities)

○ *Symptoms* (more accurately: clinical signs and/or symptoms of left ventricular hypertrophy, chronic kidney disease stages 1-4, or white matter lesions)

○ *End stage renal disease* (chronic kidney disease stage 5, dialysis or kidney transplant)

○ *Cardiac complication(s)* (atrial fibrillation, any other rhythm disturbance needing hospitalization, pacemaker or implantable cardiac defibrillator (ICD) implantation, cardiac congestion for which hospital admittance was needed, myocardial infarction, percutaneous coronary intervention or coronary artery bypass graft)

○ *Cerebrovascular accident* (stroke, as diagnosed by a neurologist)

○ *End stage renal disease and cardiac complication(s)*

○ *End stage renal disease and cerebrovascular accident*

○ *Cardiac complication(s) and cerebrovascular accident*

○ *End stage renal disease and cardiac complication(s) and cerebrovascular accident*

○ *Death*

Typically, patients progress from the asymptomatic state or acroparesthesia state to the symptoms state; from the symptoms state to a single complication state; from a single complication state to a double complication state, and from a double complication state to the triple complication state (Additional file 1 Model). In all states, patients may die. Further, patients with end stage renal disease may return to the symptomatic stage after a kidney transplant (follow-up costs of kidney transplantation were not included). The cycle length of the model was one year.

### Model data sources and assumptions

Data for the estimation of probabilities of transition to the next health state, utilities and costs (see below) were retrospectively and prospectively gathered from the Dutch Fabry cohort (S.M. Rombach et al. OJRD 2013, x:y) including 116 adults and 26 children. Among them, 75 started ERT *on indication*. Data on disease progression prior to and following the introduction of ERT were gathered from medical chart reviews. Health utility and costs data could only be gathered in the period after the introduction of ERT.

Considering data availability, the limited patient number, and the potential of confounding by indication when contrasting the treatment and no treatment situations, we made several assumptions:

– state-transition probabilities for the natural (untreated) course of Fabry disease are based on the period prior to the introduction of ERT therapy;

– ERT only decreases the probability of progressing to the next disease state;

– health utilities, health care volumes and related costs for treated as well as untreated males and females are similar for patients in the same disease state;

– no distinction was made between agalsidase alfa and agalsidase beta as evidence of superiority of either one of these products is lacking and costs are comparable for the registered dose [13-15].

### Transition probabilities

The yearly transition probabilities for the natural (untreated) course of Fabry disease were calculated by Kaplan-Meier survival analyses. The median (or less if 50% was not reached) durations to the next states were taken to calculate the transition probabilities, while accounting for the model cycle length of one year [16].

As ERT *duration in years* rather than treatment with ERT by itself affects the odds of developing major complications (from symptoms to a first complication: odds ratio (OR) 0.82 (95% CI 0.68-0.96, p=0.015); from one complication to the second complication: OR 0.52 (95% CI 0.31-0.88, p=0.014), S.M. Rombach et al. OJRD 2013, x:y), we calculated the median treatment duration in each disease state and used the odds ratio for treatment duration to calculate the relative risk reduction during that median period; subsequently, we calculated the relative risk reduction for each disease state during a single model cycle. The transition probability under ERT was then simply calculated as the yearly transition probability for untreated patients in the same disease state multiplied by 1 minus the relative risk reduction.

If, due to low patient numbers, the probability of dying during the lifetime simulation was lower in the Fabry population than the general population, we used the probability (1-probability of survival) for the general population as published by Statistics Netherlands (CBS, http://statline.cbs.nl/StatWeb/publication/?DM=SLNL&PA=37360ned&D1=0&D2=a&D3=a&D4=60&HDR=G1,T&STB=G3,G2&VW=T, survival rates, as of January 24^th^ 2011).

The validity of the yearly transition probabilities has been assessed by comparing the model results at fixed time horizons (40, 50, 60, 70 years) with literature data (available upon request) by local experts.

The yearly transition probabilities were assumed to be beta-distributed and 95% confidence intervals determined by bootstrapping (Additional file 1: Table S1-a to S1-c).

### Health outcomes

Years free of end organ damage reflected years spent in the asymptomatic, acroparesthesia or symptomatic disease states. Health status profiles were gathered quarterly with the EQ-5D quality of life questionnaire; the associated, time trade-off based health utilities [17] were averaged per patient per disease state and, subsequently, per disease state over patients. Given the cycle length of one year in the Markov model, the mean health utility equalled the number of QALYs generated during a single model cycle (Table 1).

**Table 1:** Mean health utilities per year by disease state cluster and 95% confidence intervals after boots-trapping

	N*	**Mean health utility**	**95% LCL**	**95% UCL**
Asymptomatic	19	0.874	0.804	0.934
Acroparesthesia/Symptomatic	55	0.762	0.699	0.822
Single complication	18	0.744	0.658	0.821
Multiple complications	5	0.584	0.378	0.790
Total	97	0.772	0.729	0.815

*Patients may contribute to more than one disease state.

Given low patient numbers in the more progressive disease states, results were clustered by four states: asymptomatic, acroparesthesia/symptomatic, single complication, multiple complications. LCL: lower confidence limit. UCL: upper confidence limit.

### Health care volume and costs

Costs data included the direct and indirect medical costs of health care use as well as the indirect non-medical costs of sick leave. The resource use data from the AMC were linked to available real unit costs from the AMC hospital ledger [18] (see Table 2). Unit costs were price-indexed for the year 2009.

**Table 2:** Dutch unit costs (€) for resources used

**Resource**	**Unit costs in 2009 (€)#**	**Source**
Inpatient hospital day		
AMC	596-1,036	AMC hospital ledger##
Elsewhere*	457	Dutch costing manual**
Inpatient hospital ICU day	2,183	Dutch costing manual
In-hospital day-care treatment		
AMC	274 - 845	AMC hospital ledger
Elsewhere	251	Dutch costing manual
Agalsidase α/β*** per patient per year	200,000	Report manufacturer 2010; z-index 2007
Kidney dialysis per year	60,000	[19,20]
Kidney transplantation		
first year	60,000	[19,20]
follow-up per year	not included	
Other diagnostic and therapeutic procedures	Various	AMC hospital ledger
Outpatient hospital visit		
AMC	90 - 460	AMC hospital ledger
Elsewhere*	72	Dutch costing manual
Out-of-hospital visit		
General practitioner	28	Dutch costing manual
Physiotherapist	36	Dutch costing manual
Psychiatrist/psychologist†	91.5	Dutch costing manual
Occupational physician/other††	26	AMC hospital ledger
Social worker	65	Dutch costing manual
Alternative healer	60	Expert opinion†††
Productivity loss per hour^	30	Dutch costing manual

# In case of different base years the general price index figures from the Dutch costing manual 2010 have been used to derive 2009 estimates. ## Unit costs from the AMC hospital ledger for Fabry patients include the costs of top referent health care. * Weighted unit cost based on 88% general and 12% academic inpatient days.** Unit costs from the most recent Dutch costing manual [21]. *** Weighted mean costs of therapy per patient of 70 kg per year. The costs per patient per year of full treatment amount to €198,640 for agalsidase-α and €201,346 (price-indexed for 2009) for agalsidase-β. † Weighted unit cost based on the assumption of 50%-50% distribution of visits over psychiatrists (€103) and psychologists (€80) respectively. †† Out-of-hospital visit to other care givers are assigned the lowest unit costs among the caregivers, i.e. the occupational physician. ††† The Nederlandse Mededingings Autoriteit prohibits the use of an advised tariff. Unit costs per consultation may vary considerably, depending on the type of alternative healer. As a proxy, the reported unit cost of € 60 per visit is based on an indexed derivation of the advised 2000 tariff for an acupuncturist. ^ Mean unit costs per hour across gender and age groups.

The mean yearly costs of ERT per patient were determined for a patient with an average weight of 70 kg (price-indexed for 2009: €200,503 for agalsidase alpha and €199,452 for agalsidase beta), or about €200,000 yearly. The costs of AMC hospital care were averaged per patient per disease state per year, and subsequently, the mean yearly AMC costs per disease state overall (Additional file 1: Table S2). All other used health care costs and production loss were derived from the patient by quarterly disseminated questionnaires and linked to the appropriate unit costs in Euros from the most recent Dutch costing manual (Additional file 1: Table S3 and S4). The specialist physician costs are included in the hospital costs. For each hour of production loss irrespective of gender, age, or disease state the same unit cost was applied (€30, see Table 2) (Additional file 1: Table S5). No account was given of lost productivity during working hours. Additional file 1: Table S5 shows the mean yearly costs of production loss by disease state cluster (Additional file 1: Table S5). Ethical approval was requested at the institutional review board, METC AMC. The institutional review board stated that ethical approval was not required.

### Analysis

The model was run from a lifetime perspective, starting asymptomatically at birth until the age of 70 years or death. Hypothetical cohorts of treated and untreated male and female patients were compared for both primary outcomes: costs per year without end-organ damage and costs per QALY. Incremental lifetime cost-effectiveness ratios were calculated by dividing the lifetime costs difference by the difference in lifetime years free of end-organ damage or by the difference in lifetime QALYs gained.

In the base case scenario, patients (males and females at a 1:1 ratio) entered the model at birth; ERT was initiated when symptoms developed; the costs only included the total direct and indirect medical costs; no discounting of effects or costs was performed. Univariate sensitivity analyses have been restricted to the choice of discount rate to account for time preference (discounting of effects by 1.5% and costs by 4% instead of no discounting) [22], to the unit cost of ERT per year (minus € 50,000), and to a Dutch time trade-off based health utility algorithm [23].

We performed a two-stage Monte Carlo simulation of 1,000 second-order draws from the beta-distributed yearly transition probabilities in the Markov model to represent parameter uncertainty with each single draw including 10 first-order trials to represent patient heterogeneity. For all 1,000 runs in the simulation, the net monetary benefits of ERT versus standard medical care were calculated for willingness-to-pay levels ranging from € 20,000 to € 10,000,000 per QALY with the results summarized in a cost-effectiveness acceptability curve for ERT.

In contrast with the base case scenario we additionally ran six alternative scenarios:

(1) the start of ERT in the model at the time symptoms develop is delayed until the age of 40 years, which reflects the mean age of treatment start in the Dutch Fabry cohort prior to 2010.

(2) a lower quality of life for untreated patients compared to treated patients in the same disease state with a mean difference in health utility of 0.1 [24].

(3) course of disease in patients with the classical phenotype only, without the atypical cases with a more attenuated disease course [25]. Atypical patients included patients with the R112H and P60L substitutions or patients with intermediate levels of lysoGb3 (S.M. Rombach et al. OJRD 2013, x:y).

(4) the natural course of disease in case all patients were treated with ACE-inhibitors or angiotensin-receptor blockers (which is frequent co-medication). The potential beneficial effects are extrapolated from studies in patients at high risk for cardiovascular events [26-30].

(5) no ERT for patients in the second complication groups, as previous studies have doubted the beneficial effect of ERT in more severely affected patients.

(6) adding indirect non-medical costs of production loss to the direct and indirect medical costs, assuming that these costs originate not before the 18^th^ year of a patient’s lifetime.

## Results

### Base case scenario

Over a 70 years lifetime and undiscounted, an untreated Fabry patient generates 55.0 years free of end-organ damage and 48.6 QALYs. Starting ERT in a symptomatic patient increases the number of years free of end-organ damage by 1.5 years (1.6 for males, 1.3 for females), while the number of QALYs gained increases by 1.6 QALY (1.7 for males, 1.4 for females). Table 3 shows the discounted (at 1.5%) and undiscounted incremental lifetime effects of ERT, overall and by gender.

**Table 3:** Discounted and undiscounted incremental lifetime effects of ERT versus no ERT treatment, overall and by gender (YFEOD: years free of end-organ damage; QALYs: quality adjusted life-years)

	** Discount rate 1.5%**			** Discount rate 0%**		
	**ERT**	**No ERT**	**Difference**	**ERT**	**No ERT**	**Difference**
**ALL**						
YFEOD	36.9	36.1	0.7	56.5	55.0	1.5
QALYs	32.1	31.3	0.7	50.2	48.6	1.6
**MALES**						
YFEOD	36.2	35.4	0.8	55.1	53.5	1.6
QALYs	31.7	30.9	0.8	49.5	47.8	1.7
**FEMALES**						
YFEOD	37.7	37.1	0.6	58.2	56.9	1.3
QALYs	32.6	31.9	0.7	51.1	49.7	1.4

The undiscounted costs of ERT starting in the symptomatic stage amount to €9.9 million (€9.6 M for males, €10 M for females) during a patient’s lifetime against €0.271 million (€0.273 M for males, €0.268 M for females) for standard medical care, the difference being €9.6 million (€9.3 M for males, €9.8 M for females). Table 4 shows the discounted (at 4%) and undiscounted incremental lifetime costs of ERT, overall and by gender.

**Table 4:** Discounted and undiscounted incremental lifetime total medical costs of ERT versus no ERT treatment, overall and by gender

	**Discount rate 4%**			**Discount rate 0%**		
	** ERT**	**No ERT**	**Difference**	** ERT**	**No ERT**	**Difference**
ALL	€2,504,727	€83,772	€2,420,956	€9,918.352	€270,964	€9.647,388
MALES	€2,433,824	€85,305	€2,348,519	€9,615,920	€272,892	€9,343,028
FEMALES	€2,516,273	€81,624	€2,434,649	€10,056,623	€267,517	€9,789,106

Table 5 shows the discounted and undiscounted incremental lifetime cost-effectiveness ratios (ICER), overall and by gender.

**Table 5:** Discounted and undiscounted incremental lifetime cost-effectiveness ratio’s, overall and by gender

	**Discount rate at 1.5% for effects and 4% for costs**	**Discount rate at 0% for effects and costs**
ALL		
Incremental costs per extra year free of end-organ damage	€3,318,239	€6,560,885
Incremental costs per QALY gained	€3,282,252	€6,065,529
MALES		
Incremental costs per extra year free of end-organ damage	€2,982,022	€5,917,091
Incremental costs per QALY gained	€2,947,380	€5,451,797
FEMALES		
Incremental costs per extra year free of end-organ damage	€3,797,767	€7,527,013
Incremental costs per QALY gained	€3,742,702	€6,955,612

The table shows that the ICER based on years free of end-organ damage equals €6.6 million (€5.9 M for males and €7.5 M for females); the incremental costs per QALY gained equal €6.1 million (€5.5 M for males, €7.0 M for females).

### Scenario analyses

Table 6 shows the discounted and undiscounted incremental lifetime ICERs for years free of end-organ damage and for QALYs respectively under different scenarios.

**Table 6:** Scenario-analyses*: discounted and undiscounted incremental lifetime cost- effectiveness ratios

	**ICER based on years free of end-organ damage, in €**		**ICER based QALYs, in €**	
	**Discounted**	**Undiscounted**	**Discounted**	**Undiscounted**
Base case typical patient	3,318,239	6,560,885	3,282,252	6,065,529
1. start ERT at 40 years	3,662,891	12,996,662	2,158,245	7,637,076
2. lower QoL during the natural course	-	-	509,719	1,226,674
3. course of disease in patients with the classical phenotype only	3,274,869	6,280,356	3,015,385	5,575,064
4. ACE-ARB during natural course	293,213,929	566,675,324	11,559,105	21,223,686
5. No ERT in case of 2 complications	3,307,363	6,529,644	3,271,494	6,036,644
6. including indirect costs of production loss	3,320,374	6,568,971	3,284,265	6,073,006

* Given a 1:1 male to female ratio.

If ERT is not initiated before the age of 40, both, lifetime costs and effects decrease, but to a different extent, resulting in increased undiscounted ICERs, especially in case of the incremental costs per extra year free of end-organ damage. If ERT not only slows disease progression, but also results in a higher health utility of 0.1 when treated, the undiscounted ICER based on QALYs drops by 80% to €1.2 million. ERT in classically affected patients only results in 5% to 8% lower ICERs compared to the base case. The impact of additional treatment with ACE-ARB minimizes the value of ERT as is demonstrated by the increased ICERs compared with the base case scenario. The final two scenarios, not treating patients with more advanced disease or adding the indirect costs of production loss to the total medical costs, marginally affect the ICERs. In all scenarios, the ICERs were more favourable for males than for females (data not shown).

### Sensitivity analyses

Differential discounting of effects (at 1.5%) and costs (at 4%) improves the undiscounted ICERs for the base case scenario as well as for scenarios 3 to 6 by 48-49% in case of years free of end-organ damage and by 45-46% in case of QALYs. Application of discounting in the scenario start ERT at the age of 40 decreases the undiscounted ICERs by 72%. Discounting decreases the incremental costs per extra QALY in the scenario with a lower health utility when untreated by 58%.

A 25% reduction of the yearly cost of ERT from €200,000 to €150,000 demonstrates the dominance of this cost component: the incremental cost-effectiveness ratios decrease by a similar margin. For instance, the extra costs per additional QALY for a typical patient, a male patient and a female patient amount to €4,549,987, €4,089,674 and €5,217,573 respectively against €6.1 million, €5.5 million and €7.0 million in the base case scenario.

Mean health utilities by disease state based on preferences from the Dutch general populations are non-significantly higher than the UK based data [17], with lower losses in health utility during disease progression. Hence, slowing disease progression results in less QALYs to be gained, if Dutch preferences were to be used instead. Consequently, the ICERs lie above the UK based values reported in this paper.

**Figure 1:**
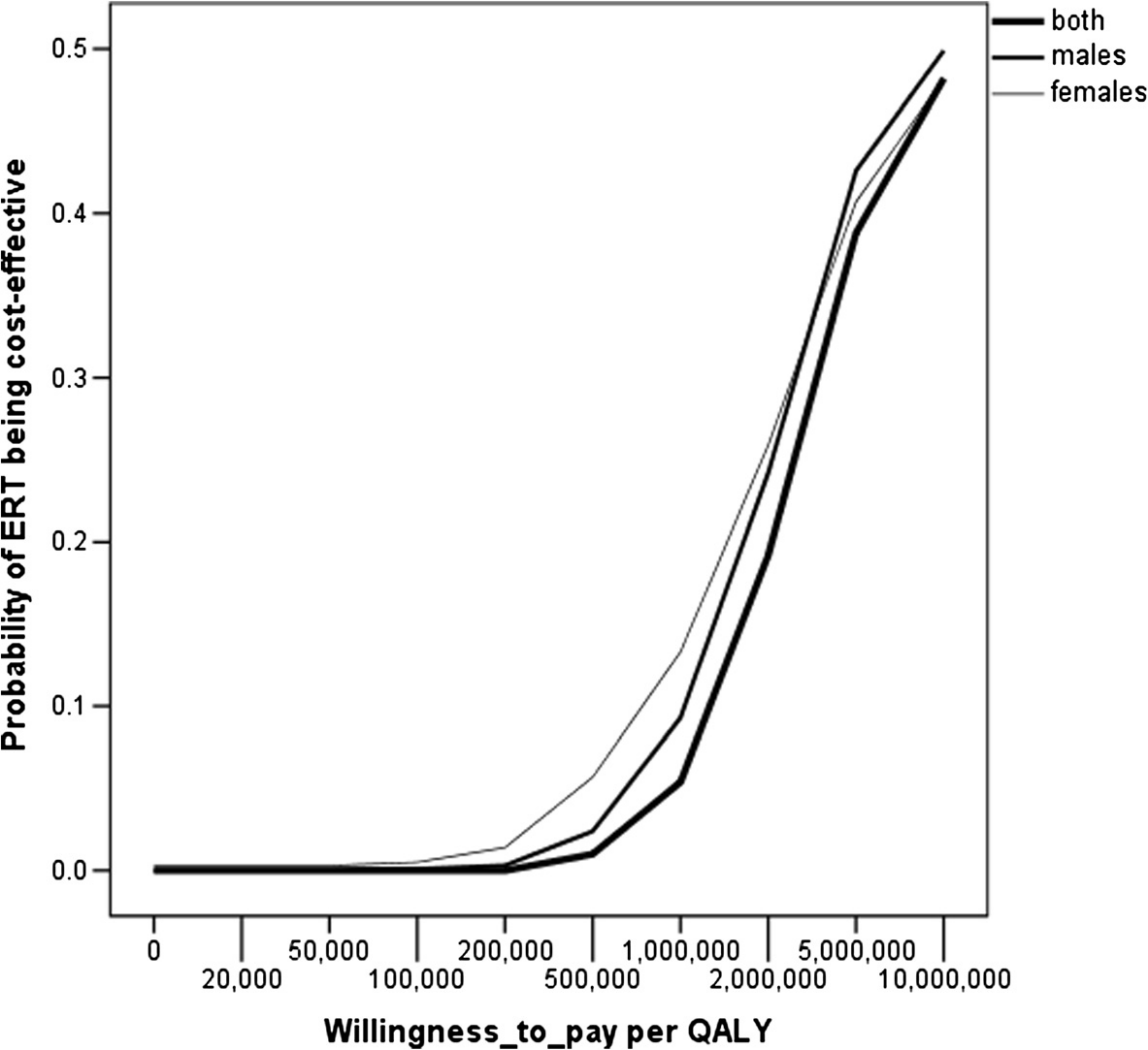
**Cost-effectiveness acceptability curves of ERT versus no treatment for various levels of willingness-to-pay per QALY after 1,000 runs in a Monte Carlo simulation of beta-distributed state transition probabilities.** The proportions of net monetary benefits larger than zero for these willingness-to-pay levels represent the probabilities of ERT being cost-effective in comparison with standard medical care and are reported in the cost-effectiveness acceptability curve.

Figure 1 shows the cost-effectiveness acceptability curves of ERT against standard medical care for various levels of the willingness-to-pay per QALY and in absence of discounting. It shows that the probability of ERT being an efficient intervention is near zero when more conventional willingness-to-pay values up to €100,000 are considered and does not exceed 0.5 for values below €10 million.

## Discussion

### Summary of major findings

The model demonstrated small gains in effectiveness with 1.5 extra years free of end-organ damage and 1.6 QALYs gained (or both 0.7, discounted) for treatment with ERT. The extra lifetime costs of ERT compared with standard medical care amounted to €9.65 million per patient (or €2.42 million, discounted). The related incremental cost-effectiveness ratios ranged from €3.2 million (discounted) to €6.5 million (no discounting).

The sensitivity and scenario analyses revealed that these cost-effectiveness ratios could be substantially reduced by lowering the high costs of the drug itself (near proportional impact) or a modest health gain of 0.1 QALY per year in treated patients (minus 80%). To a lesser extent, patient selection optimizes the ICERs, e.g. classically affected patients with a more severe disease course [25].

The probabilistic sensitivity analysis showed that the uncertainties concerning the estimated transition probabilities only affect health care policy making substantially as of willingness-to-pay values of €1 million per QALY or above.

On the other hand, the additional value of ERT could easily be nullified, if ACE-ARB for the reduction of proteinuria reduces the risk on cardiovascular complications equally well in Fabry patients as in other high risk populations [31,32]. This is mostly explained by the assumption that treatment with ACE-ARB alone would be as effective as ERT for risk reduction of a first complication (see Additional file 2). Whether this assumption is valid, is a research area of interest.

The most cost-effective scenario is early treatment of a male patient with a classical phenotype and discarding additional beneficial effect of ACE-ARB. In contrast, in milder affected patients, such as women and atypical phenotypes, the gain in QALYs with ERT is only modest. At equal costs of treatment, cost-effectiveness is consequently less. It needs to be emphasized that differentiating by gender is an oversimplification: some females may be as severely affected as males and thus could equally benefit from therapy.

Our assumption that the health utilities, health care volume and the related costs other than the costs of ERT medication itself were similar for treated and untreated patients in the same disease state should be considered as a study limitation. The scenario of a lower health utility during the natural course demonstrated that this may lead to an underestimation of the value of ERT. Actually, it would be of the utmost importance to accurately assess the difference in health utility between patients on ERT and patients not on ERT in the “real world”, without being hampered by confounding by indication. Unfortunately, there is a lack of opportunity in this respect.

Another study limitation was the exclusion of costs of follow-up related to a kidney transplant in particular; only the costs of a kidney transplant during its first year were incorporated. Including these follow-up costs would have increased the structural complexity of the memory-less Markov model considerably without – in view of the small number of Dutch Fabry patients receiving a kidney transplant - meaningful consequences in terms of health policy.

When comparing the treatment effect as determined in the Dutch Fabry cohort to the results of the only randomized-controlled trial on development of end-organ complications available, we notice a similar risk reduction (for one complication, developing a second complication) [32]. This supports the validity of the estimated treatment effect used in the model.

### Implications and recommendations for future studies

The present study highlights the enormous costs associated with orphan drugs as was recently also pointed out by others [8,9]. Compared to previously reported international data on costs per QALY, ranging from US $300,000 to UK £250,000 per QALY, the present results should put (i) the high unit costs of ERT itself, (ii) the process of drug development with perhaps a lack of sufficient early assessment of a drug’s clinical potential, and (iii) the cooperation between manufacturers and governmental agencies on the agenda.

As ERT itself is costly, the ICERs are reported in millions and are not coming near to thresholds that might be considered affordable from a societal perspective [33]. Recently, a health technology assessment in the UK showed that at least 3.6 discounted QALYs for an adult patient with Fabry disease are needed each year for ERT to be cost-effective, considering a willingness-to-pay of £30 000 [34]. Based on the incremental lifetime total medical costs data generated by the Markov model (see Table 4), a willingness-to-pay per QALY in the Netherlands of €80 000, and a treatment window of about 46 years between the onset of symptoms and death in the natural history cohort, at least 0.65 discounted and 2.6 undiscounted QALYs would have to be generated each year under ERT treatment. It is clear that such yearly gain is unrealistic, which makes it understandable, if one considers performing a cost-effectiveness or cost-utility analysis infeasible [34].

Based on the data on disease progression while simultaneously aiming for cost containment, new therapeutic guidelines should therefore be developed to differentiate patients who may benefit from ERT from those who probably will not. Incorporating findings on associations between ERT and key disease progression markers in Fabry disease from the abovementioned health technology assessment in the UK, especially with regard to cardiac and renal manifestations, may prove highly valuable in this respect [34].

At this time no data exist that support the idea of agalsidase alfa being superior to agalsidase beta, or vice-versa. It was beyond the scope of this study to address this.

Pre-symptomatic initiation of ERT may improve cost-effectiveness by prevention of complications that impact on quality of life but there are no data available yet to evaluate this scenario.

## Conclusion

In conclusion, this study showed that the affordability of ERT of Fabry disease remains at stake. The modest effectiveness drives the costs per QALY and even the costs per year free of end-organ damage to numbers expressed in millions of euros. New therapeutic guidelines should be developed to differentiate high responders from low or no responders to ERT, diagnostic procedures should be improved, and the add-on value of ERT relative to the effect of ACE-ARB should be assessed.

## Competing interests

CEH and GEL received reimbursement of expenses and honoraria for lectures on the management of lysosomal storage diseases from Genzyme Corporation, Shire, Actelion and Amicus Therapeutics. All honoraria are donated to the Gaucher Stichting, a national foundation that supports research in the field of lysosomal storage disorders. SMR and MGD declare that they have no competing interests.

## Authors’ contributions

CEM and MGD initiated the study. SMR coordinated the study. SMR and GEL participated in the data collection. All authors were involved in the design of the study. SMR, CEM and MGD contributed to the analysis. All authors were involved in writing the report. All authors read and approved the final manuscript.

## Funding

This study was supported by a grant from the Ministry of Health (ZonMW). Researchers worked independently from the funders. The funding source had no involvement in study design; in the collection, analysis, and interpretation of data; in the writing of the report; and in the decision to submit an article for publication.

## Supplementary Material

Additional file 1 **Model Markov model for Fabry disease.** Model structure for bottom half (ERT treatment) is identical. In all Markov states, patients may die (not shown). **Table S1-a.** Yearly beta-distributed state-transition probabilities for untreated and treated males. **Table S1-b.** Yearly beta-distributed state-transition probabilities for untreated and treated females. **Table S1-c.** Yearly beta-distributed state-transition probabilities for untreated and treated males and females. **Table S2.** Mean yearly numbers and costs of diagnostic and therapeutic procedures* in the AMC. Clustering of disease states was necessary because of low patient numbers. LCL: lower confidence limit. UCL: upper confidence limit. **Table S3.** Mean yearly numbers and costs of (ICU) inpatient days in hospitals other than the AMC. Costs were derived from the quarterly disseminated patient questionnaires and averaged per patient per disease state cluster, multiplied by four to arrive at yearly mean estimates per patient per disease state cluster, and subsequently, averaged per disease state cluster. LCL: lower confidence limit. UCL: upper confidence limit. **Table S4.** Mean yearly numbers and costs of various out-of-hospital consultations by disease state cluster. First, costs per individual patient per disease state per year were calculated; subsequently, the total average per patient per disease state was calculated. LCL: lower confidence limit. UCL: upper confidence limit. **Table S5.** Mean yearly indirect costs of sick leave by disease state cluster. The overall mean number of working hours per working day and overall mean number of working days per week for patients with paid work were calculated. For each patient with a paid job the individual mean volume of sick leave in days per fortnight was calculated over available repeated measurements and per disease state. The resulting individual mean volume was multiplied by 26 and by the overall mean number of hours per working day for patients with a paid job to arrive at yearly mean production loss estimates per patient per disease state. For patients with a permanent sick leave because of Fabry disease a yearly volume of production loss was defined based on the overall mean number of working hours per working day and overall mean number of working days per week for patients with paid work. For patients without paid work for reasons other than Fabry disease a zero volume of production loss was assumed. LCL: lower confidence limit. UCL: upper confidence limit. Click here for file

Additional file 2 **APPENDIX.** The cost-effectiveness and cost-utility model. Click here for file
