# Supplementary material for: Cost-effectiveness of enzyme replacement therapy for Fabry disease
Source: Orphanet J Rare Dis. 2013 Feb 19;8:29. doi: 10.1186/1750-1172-8-29 (PMC3598841; doi:10.1186/1750-1172-8-29)
Supplement: Additional file 1 — Model Markov model for Fabry disease. Model structure for bottom half (ERT treatment) is identical. In all Markov states, patients may die (not shown). Table S1-a. Yearly beta-distributed state-transition probabilities for untreated and treated males. Table S1-b. Yearly beta-distributed state-transition probabilities for untreated and treated females. Table S1-c. Yearly beta-distributed state-transition probabilities for untreated and treated males and females. Table S2. Mean yearly numbers and costs of diagnostic and therapeutic procedures* in the AMC. Clustering of disease states was necessary because of low patient numbers. LCL: lower confidence limit. UCL: upper confidence limit. Table S3. Mean yearly numbers and costs of (ICU) inpatient days in hospitals other than the AMC. Costs were derived from the quarterly disseminated patient questionnaires and averaged per patient per disease state cluster, multiplied by four to arrive at yearly mean estimates per patient per disease state cluster, and subsequently, averaged per disease state cluster. LCL: lower confidence limit. UCL: upper confidence limit. Table S4. Mean yearly numbers and costs of various out-of-hospital consultations by disease state cluster. First, costs per individual patient per disease state per year were calculated; subsequently, the total average per patient per disease state was calculated. LCL: lower confidence limit. UCL: upper confidence limit. Table S5. Mean yearly indirect costs of sick leave by disease state cluster. The overall mean number of working hours per working day and overall mean number of working days per week for patients with paid work were calculated. For each patient with a paid job the individual mean volume of sick leave in days per fortnight was calculated over available repeated measurements and per disease state. The resulting individual mean volume was multiplied by 26 and by the overall mean number of hours per working day for patients with a paid job to arrive at y [file 1750-1172-8-29-S1.doc]

**Supplement 1**


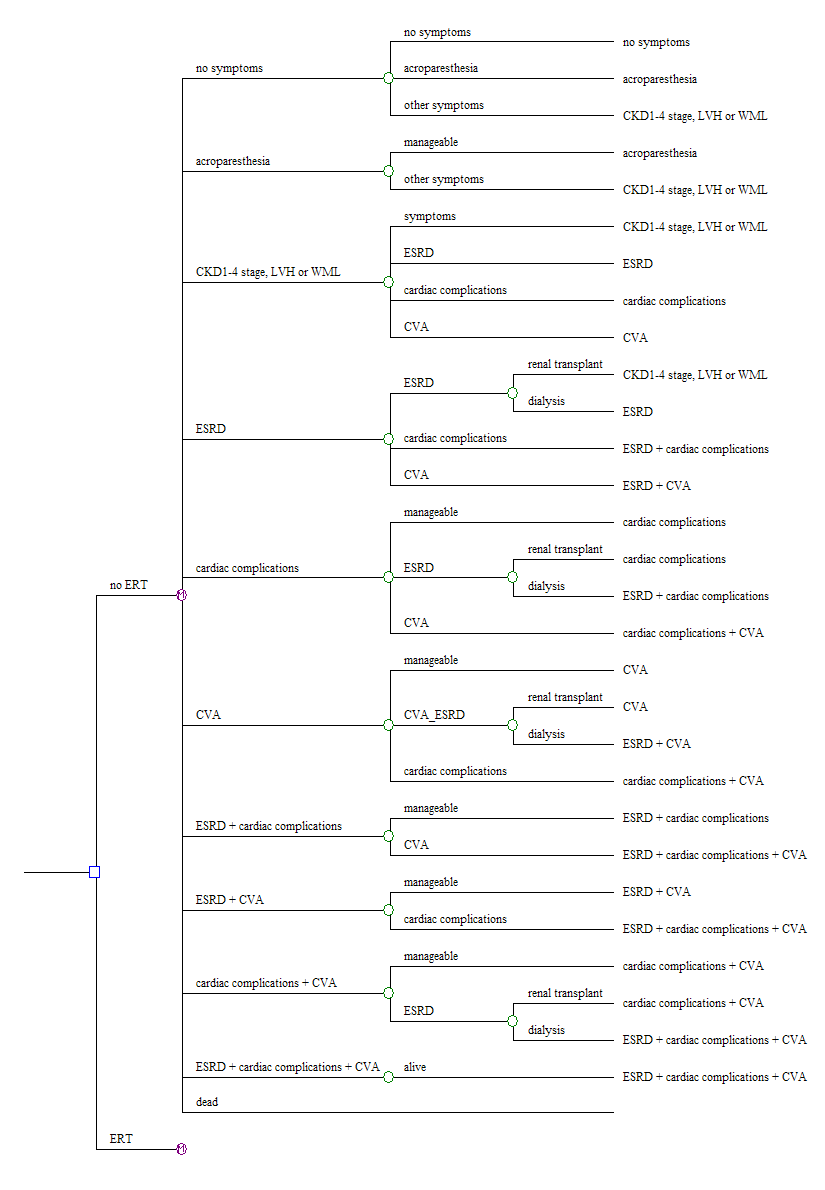


**Supplement 1. Markov model for Fabry disease.** Model structure for bottom half (ERT treatment) is identical. In all Markov states, patients may die (not shown).

**Supplement 2-**a. Yearly beta-distributed state-transition probabilities for untreated and treated males

| Transition | Yearly probability  natural course in males | 95% CI | Yearly probability  ERT in males | 95% CI |
| --- | --- | --- | --- | --- |
| No symptoms > Acroparesthesia | 0.0830 | 0.0027-0.2852 | 0.0830 | 0.0035-0.2642 |
| Acroparesthesia > symptoms | 0.0711 | 0.0019-0.2354 | 0.0711 | 0.0020-0.2409 |
| Acroparesthesia > death | * | * | * | * |
| No symptoms > symptoms | 0.0391 | 0.0008-0.1432 | 0.0391 | 0.0008-0.1432 |
| Symptoms > ESRD | 0.0020 | 0.0000-0.0076 | 0.0017 | 0.0000-0.0059 |
| Symptoms > CC | 0.0097 | 0.0003-0.0354 | 0.0085 | 0.0002-0.0324 |
| Symptoms > CVA | 0.0034 | 0.0001-0.0127 | 0.0029 | 0.0001-0.0108 |
| Symptoms > death | 0.0006 | 0.0000-0.0021 | 0.0006 | 0.0000-0.0022 |

*CBS: mortality data based on the general population were used for this disease stage

**Supplement 2-**b. Yearly beta-distributed state-transition probabilities for untreated and treated females

| Transition | Yearly probability  natural course in females | 95% CI | Yearly probability  ERT in females | 95% CI |
| --- | --- | --- | --- | --- |
| No symptoms > Acroparesthesia | 0.0472 | 0.0008-0.1662 | 0.0472 | 0.0012-0.1620 |
| Acroparesthesia > symptoms | 0.1018 | 0.0025-0.3781 | 0.1018 | 0.0028-0.3216 |
| Acroparesthesia > death | * | * | * | * |
| No symptoms > symptoms | 0.0391 | 0.0009-0.1341 | 0.0391 | 0.0009-0.1251 |
| Symptoms > ESRD | 0.0018 | 0.0000-0.0072 | 0.0016 | 0.0000-0.0065 |
| Symptoms > CC | 0.0071 | 0.0001-0.0275 | 0.0062 | 0.0002-0.0268 |
| Symptoms > CVA | 0.0027 | 0.0001-0.0097 | 0.0024 | 0.0001-0.0093 |
| Symptoms > death | * | * | * | * |

*CBS: mortality data based on the general population were used for this disease stage

**Supplement 2-**c. Yearly beta-distributed state-transition probabilities for untreated and treated males and females

| Transition | Yearly probability  natural course | 95% CI | Yearly probability  ERT | 95% CI |
| --- | --- | --- | --- | --- |
| ESRD > CC | 0.0133 | 0.0004-0.0462 | 0.0086 | 0.0002-0.0316 |
| ESRD > CVA | 0.0098 | 0.0002-0.0344 | 0.0063 | 0.0002-0.0260 |
| ESRD > death | 0.0169 | 0.0004-0.0648 | 0.0109 | 0.0003-0.0425 |
| CC > ESRD | 0.0077 | 0.0003-0.0316 | 0.0050 | 0.0001-0.0186 |
| CC > CVA | 0.0118 | 0.0006-0.0526 | 0.0077 | 0.0002-0.0285 |
| CC > death | 0.0206 | 0.0008-0.0706 | 0.0134 | 0.0003-0.0519 |
| CVA > ESRD | 0.0070 | 0.0002-0.0266 | 0.0045 | 0.0001-0.0168 |
| CVA > CC | 0.0146 | 0.0003-0.0620 | 0.0094 | 0.0002-0.0321 |
| CVA > death | 0.0186 | 0.0005-0.0655 | 0.0120 | 0.0003-0.0397 |
| 2 complications > 3^rd^ C | 0.1379 | 0.0167-0.3565 | 0.1379 | 0.0216-0.3506 |
| 2 complications > death | 0.4068 | 0.1438-0.7065 | 0.4068 | 0.1512-0.7009 |
| 3 complications > death | 0.4068 | 0.1228-0.6943 | 0.4068 | 0.1327-0.6961 |

| **Supplement 3. Mean yearly numbers and costs of diagnostic and therapeutic procedures* in the AMC.** Clustering of disease states was necessary because of low patient numbers. LCL: lower confidence limit. UCL: upper confidence limit. | | | | | | | |
| --- | --- | --- | --- | --- | --- | --- | --- |
|  | Mean costs per procedure in € | Number of procedures | 95% LCL | 95% UCL | Costs in € | 95% LCL | 95% UCL |
|  |  |  |  |  |  |  |  |
| Asymptomatic (N=21) | 24.5 | 54.4 | 43.2 | 67 | 1,334 | 889 | 1,832 |
| Symptomatic / acroparesthesia (N=71) | 32.8 | 96.5 | 88.1 | 104.7 | 3,167 | 2,613 | 3,713 |
| Single complication (N=18) | 26.1 | 117.9 | 104.9 | 130.4 | 3,073 | 2,428 | 3,864 |
| Multiple complications (N=4) | 46.8 | 138.1 | 100 | 172 | 6,466 | 2,358 | 10,679 |
|  |  |  |  |  |  |  |  |
| Total (N=114) | 31.3 | 93.6 | 86.5 | 101.1 | 2,930 | 2,513 | 3,379 |

*AMC inpatient days too were counted as procedures here.

| **Supplement 4. Mean yearly numbers and costs of (ICU) inpatient days in hospitals other than the AMC .** Costs were derived from the quarterly disseminated patient questionnaires and averaged per patient per disease state cluster, multiplied by four to arrive at yearly mean estimates per patient per disease state cluster, and subsequently, averaged per disease state cluster. LCL: lower confidence limit. UCL: upper confidence limit. | | | | | | |  |
| --- | --- | --- | --- | --- | --- | --- | --- |
|  | Mean costs per day € | Number of (ICU) inpatient days | 95% LCL | 95% UCL | Costs in € | 95% LCL | 95% UCL |
|  |  |  |  |  |  |  |  |
| Asymptomatic (N=19) | - | 0 | - | - | 0 | - | - |
| Symptomatic/ acroparesthesia (N=56) | 454.5 | 0.11 | 0 | 0.24 | 50 | 0 | 110 |
| Single complication (N=18) | 572.7 | 4.98 | 1.26 | 9.44 | 2,852 | 643 | 5,581 |
| Multiple complications (N=5) | 827.6 | 3.73 | 0 | 8 | 3,087 | 0 | 7,090 |
|  |  |  |  |  |  |  |  |
| Total (N=98) | 606.8 | 1.17 | 0.41 | 2.1 | 710 | 224 | 1278 |

| **Supplement 5. Mean yearly numbers and costs of various out-of-hospital consultations by disease state cluster.** First, costs per individual patient per disease state per year were calculated; subsequently, the total average per patient per disease state was calculated. LCL: lower confidence limit. UCL: upper confidence limit. | | | | | | | |
| --- | --- | --- | --- | --- | --- | --- | --- |
|  |  |  | |  |  |  |  |
|  | Volumes | 95% LCL | 95% UCL |  | Costs in € | 95% LCL | 95% UCL |
| **Asymptomatic (N=19)** |  |  |  |  |  |  |  |
| General physician | 2.1 | 1.4 | 2.9 |  | 59 | 38 | 81 |
| Physiotherapist | 5.4 | 1.2 | 10.5 |  | 194 | 42 | 378 |
| Psychologist/psychiatrist | 3.7 | 0.0 | 8.3 |  | 339 | 0 | 787 |
| Company physician | 0.0 | . | . |  | 0 | . | . |
| Social worker | 0.2 | 0.0 | 0.5 |  | 11 | 0 | 32 |
| Alternative healer | 0.2 | 0.0 | 0.6 |  | 13 | 0 | 40 |
| Other | 1.5 | 0.3 | 2.9 |  | 38 | 9 | 76 |
| Total | 13.0 | 6.3 | 20.7 |  | 654 | 255 | 1,103 |
| **Symptomatic/ acroparesthesia (N=56)** |  |  |  |  |  |  |  |
| General physician | 3.5 | 2.2 | 4.9 |  | 97 | 61 | 138 |
| Physiotherapist | 5.6 | 2.4 | 9.4 |  | 201 | 87 | 340 |
| Psychologist/psychiatrist | 1.5 | 0.2 | 3.3 |  | 136 | 17 | 298 |
| Company physician | 0.2 | 0.1 | 0.4 |  | 6 | 3 | 10 |
| Social worker | 0.3 | 0.0 | 0.7 |  | 21 | 0 | 47 |
| Alternative healer | 0.5 | 0.1 | 0.9 |  | 27 | 5 | 54 |
| Other | 1.1 | 0.2 | 2.2 |  | 27 | 6 | 57 |
| Total | 12.6 | 7.8 | 18.0 |  | 516 | 297 | 771 |
| **Single complication N=18)** |  |  |  |  |  |  |  |
| General physician | 3.7 | 2.1 | 5.8 |  | 104 | 58 | 161 |
| Physiotherapist | 18.5 | 4.8 | 36.7 |  | 667 | 172 | 1,321 |
| Psychologist/psychiatrist | 0.1 | 0.0 | 0.4 |  | 13 | 0 | 40 |
| Company physician | 0.3 | 0.0 | 0.6 |  | 7 | 0 | 15 |
| Social worker | 0.4 | 0.0 | 0.8 |  | 24 | 0 | 51 |
| Alternative healer | 0.2 | 0.0 | 0.7 |  | 13 | 0 | 40 |
| Other | 3.1 | 0.3 | 7.5 |  | 81 | 8 | 195 |
| Total | 26.4 | 12.1 | 44.6 |  | 910 | 393 | 1,551 |
| **Multiple complications (N=5)** | |  |  |  |  |  |  |
| General physician | 4.8 | 0.0 | 10.0 |  | 134 | 0 | 280 |
| Physiotherapist | 8.8 | 0.0 | 18.7 |  | 317 | 0 | 673 |
| Social worker | 0.3 | 0.0 | 0.7 |  | 17 | 0 | 43 |
| Total | 13.9 | 4.0 | 24.5 |  | 469 | 112 | 863 |
| **Total (N=98)** |  |  |  |  |  |  |  |
| General physician | 3.3 | 2.5 | 4.2 |  | 93 | 70 | 119 |
| Physiotherapist | 8.1 | 4.8 | 12.3 |  | 291 | 173 | 442 |
| Psychologist/psychiatrist | 1.6 | 0.4 | 3.1 |  | 146 | 38 | 279 |
| Company physician | 0.2 | 0.1 | 0.3 |  | 5 | 2 | 8 |
| Social worker | 0.3 | 0.1 | 0.6 |  | 19 | 7 | 36 |
| Alternative healer | 0.3 | 0.1 | 0.6 |  | 21 | 7 | 37 |
| Other | 1.5 | 0.6 | 2.6 |  | 38 | 15 | 69 |
| Total | 15.3 | 11.1 | 20.3 |  | 613 | 425 | 823 |

**Supplement 6. Mean yearly indirect costs of sick leave by disease state cluster.** The overall mean number of working hours per working day and overall mean number of working days per week for patients with paid work were calculated. For each patient with a paid job the individual mean volume of sick leave in days per fortnight was calculated over available repeated measurements and per disease state. The resulting individual mean volume was multiplied by 26 and by the overall mean number of hours per working day for patients with a paid job to arrive at yearly mean production loss estimates per patient per disease state. For patients with a permanent sick leave because of Fabry disease a yearly volume of production loss was defined based on the overall mean number of working hours per working day and overall mean number of working days per week for patients with paid work. For patients without paid work for reasons other than Fabry disease a zero volume of production loss was assumed. LCL: lower confidence limit. UCL: upper confidence limit.

|  | N | Costs of production loss in €* | 95% LCL | 95% UCL |
| --- | --- | --- | --- | --- |
| Asymptomatic | 19 | 258 | 64 | 502 |
| Symptomatic/acroparesthesia | 56 | 6,001 | 2,986 | 9,272 |
| Single complication | 18 | 7,166 | 2,168 | 14,197 |
| Multiple complications | 5 | 28,436 | 0 | 47,393 |
| Total | 98 | 6,246 | 3,838 | 9,402 |

* Based on the human capital valuation method. Volume data in number of hours can be

derived by dividing the cost figures by the unit cost per lost working hour (or €30).
