# Supplementary material for: Cost-effectiveness of enzyme replacement therapy for Fabry disease
Source: Orphanet J Rare Dis. 2013 Feb 19;8:29. doi: 10.1186/1750-1172-8-29 (PMC3598841; doi:10.1186/1750-1172-8-29)
Supplement: Additional file 2 — APPENDIX. The cost-effectiveness and cost-utility model. [file 1750-1172-8-29-S2.doc]

**APPENDIX. The cost-effectiveness and cost-utility model.**

This appendix contains the technical background and the accountability of the probabilities used in the design and construction of the Markov state-transition cost-effectiveness model.

*Abbreviations and definitions used in the description*

CC: cardiac complications; definition for AMC data: onset of atrial fibrillation, onset of any other rhythm disturbance needing hospitalization, pacemaker or implantable cardiac defibrillator (ICD) implantation, congestive heart failure for which hospital admittance was needed, myocardial infarction, percutaneous coronary intervention, or coronary artery bypass graft. In some literature studies the definition of cardiac complications also included angina.

Markov Correction factor: Yearly transition probability = 1-(1-tp_t_) ^1/t^ in which tp_t_ is overall probability over time period t.

CKD stages: chronic kidney disease stages

Clinical events: ESRD, CVA or cardiac complications/event

CVA: cerebrovascular accident, as diagnosed by a neurologist

ESRD: End stage renal disease, requiring dialysis or kidney transplant (Tx)

LVH: left ventricular hypertrophy

NH: natural history

Symptoms: presence of any of the following symptoms: left ventricular hypertrophy (LVH), CKD1-4, white matter lesion(s).

WML: white matter lesion(s)

**1. Aim of the Markov-analysis**

The yearly costs without end-organ damage are the primary outcome measure for the cost-effectiveness analysis. The costs per quality adjusted life-year (QALY) are the primary outcome measure for the cost-utility analysis. A life-time Markov-model is constructed to include the longer term consequences of treatment.

**2. Methods**

**Model overview**

A Markov state-transition model was built, allowing the differentiation of the consecutive phases of Fabry disease. The model was programmed in Tree Age 3.5. All analyses and reports followed the existing guidelines for health technology assessment [1, 2] and decision analytic cost-effectiveness modeling [3].

**2.1. Transition states**

It is expected that progression to end-organ damage has the most important impact on quality of life (the utility), leading to increase of health care and non-health care costs which might be prevented by timely intervention. With the life-time Markov model the yearly costs without end-organ damage, as well as the costs per quality adjusted life year are assessed. For that purpose the model consists of 11 mutually exclusive disease states. The disease states are as follows: no symptoms (no acroparesthesia, white matter lesions, CKD1-4 or LVH present), acroparesthesia, symptoms (defined as white matter lesions, CKD1-4 or LVH present) and the complications ESRD, one or more cardiac complications (CC) or CVA and the combination of two or three complications, all as previously defined and shown in table 1. Finally, a state of death was included. In case a patient develops acroparesthesia or any of the other symptoms or disease complications, there is an indication for treatment with enzyme replacement therapy.

The model is designed to depict the prevalence and progression of disease symptoms and complications in a simulated cohort of patients with Fabry disease. The model shows a patient’s medical outcome, utility and costs per disease state, from the day of birth, until the age of 70 years or death. The length of one cycle represents 1 year.

**Table 1. Transition states in the Markov model.**

| Disease states | |
| --- | --- |
| No symptoms | no LVH, CKD1-4 or wml |
| Acroparesthesia | acroparesthesia |
| Symptoms | LVH, CKD1-4 or white matter lesions |
| End stage renal disease (ESRD) | CKD5, dialysis or kidney transplant |
| Cardiac complication(s) (CC) | atrial fibrillation, any other rhythm disturbance needing hospitalization, pacemaker or implantable cardiac defibrillator (ICD) implantation, cardiac congestion for which hospital admittance was needed, myocardial infarction, percutaneous coronary intervention or coronary artery bypass graft |
| Cerebrovascular accident (CVA) | Stroke, as diagnosed by a neurologist |
| ESRD + CC | ESRD and one or more cardiac complications |
| ESRD+CVA | ESRD and one or more strokes |
| CC+CVA | One or more cardiac complications and one or more strokes |
| ESRD+CC+CVA | ESRD, one or more cardiac complications and one or more strokes |
| Dead | Death |

*Patients*

*Symptoms*

The patients in the symptomatic group, including 100 patients (48 males, 52 females) with a confirmed diagnosis of Fabry disease are described elsewhere (Rombach et al manuscript included).Because of current criteria for start of ERT, patients without symptoms were not treated, with the exception of 4 patients (2 males and 2 females) who presented with acroparesthesia only. Acroparesthesia is a criterium for start of treatment but was not evaluated as predictive symptom for development of complications in the Markov model. There were few data on patients that had no symptoms at baseline and developed symptoms during follow-up (n=7, 2 males and 5 females). In addition, most patients were already known with symptoms before they were referred to the AMC outpatient clinic. Therefore age of the first presentation of acroparesthesia was estimated in the current pediatric cohort.

*Complications: development of ESRD, a cardiac complication, CVA, or death.*

The patients in the complication group, including 33 patients (21 males, 12 females) are described elsewhere (Rombach et al manuscript included).

**2.2 Transition probabilities**

The Markov state-transition model was developed with data from the Dutch Fabry registry, validated with literature data and evaluated by expert opinion. Yearly state-transition probabilities were calculated from data from the entire Dutch Fabry cohort. This cohort consisted of all registered Fabry disease patients in the Netherlands with a diagnosis of Fabry disease. Data of these patients were prospectively collected from the moment that ERT was available (from 1999). Inclusion of new patients with Fabry disease started at the beginning of the study (October 2008) up to August 2010. Prospective data were collected up to December 31^st^ 2010. Complete follow-up data (date of birth, gender, development of clinical events and the age at the time of the clinical events) were available of 142 Fabry patients including all pediatric patients. The yearly transition probabilities for the natural (untreated) course of Fabry disease were calculated by Kaplan-Meier survival analyses, using retrospective data only.

For the analyses of the effect of ERT compared to no treatment on the transition probabilities, a distinction was made between the natural history cohort and the ERT cohort (Rombach et al manuscript included). For the analyses of the probabilities to the next disease state as visualized in the Markov decision tree, patients in the previous state were selected for calculation of the transition probability to the next state. For example, the group with symptoms was studied to calculate the rate of development of the first complication,.This implies that there is always a fixed progression of disease states from asymptomatic, through a symptomatic state and from the acroparesthesia state through a symptomatic state towards complication(s).

The yearly transition probabilities and the relative risk reduction of treatment were calculated as follows: the odds for developing a first or second complication and the contribution of age, gender and ERT duration was assessed (Rombach et al manuscript included); as in a Markov model patients enter disease states at different time points and therefore age and duration of treatment may differ, a constant yearly probability independent of age and duration of ERT was estimated. For untreated as well as treated patients, the time to the next disease state was calculated by using Kaplan-Meier analysis when 50% (or less if 50% was not reached) of the cohort in a certain state had reached the next state. Then the yearly probability was calculated by dividing the cumulative proportion (i.e. 0.50) by the median time of follow-up. These probabilities were corrected for the conditional nature of transition probabilities in the cyclic Markov model with a lifetime horizon (the Markov Correction factor: transition probability (tp)_1_ = 1 - (1 - tp_t_)^1/t^ where tp_t_ is the overall probability over time period t , see [4]. Finally the distribution of each type of complication (ESRD, CC, CVA or death) was assessed within the first, second and third complication for males and females separately when applicable and the yearly transition probability of developing a complication was multiplied with the proportion of each type of complication. For calculation of the treatment effect on the course of disease in case of initiation of ERT, the median treatment duration in each disease state of the ERT treated cohort was calculated and the odds ratio was used to calculate the relative risk reduction during that period. Then the yearly relative risk reduction was calculated, using the Markov cycle correction [4]. The yearly probability in case of ERT initiation was simply calculated as the yearly probability in the same disease state during the natural course multiplied by 1-relative risk reduction.

**Yearly probabilities for mortality**

For the mortality rate, the yearly probability of death in the different transition states was calculated as described above. The yearly probability of dying in the Fabry population was compared to the death rate of the healthy population (www.cbs.nl, survival data, as of January 24th).and in case the probability of dying in any disease state was lower in the Fabry population as compared to the healthy population (for example, patients in a disease state were young or in case of prolonging of life due to treatment effects) the background mortality was used based on the yearly survival rates (1-probability of survival).

**2.3 Utilities**

Yearly utility values were calculated from EQ-5D questionnaire completed by the majority of the patients in the Fabry cohort.

**2.4. Costs**

Yearly costs were estimated per each individual patient who also completed a questionnaire developed for this purpose. Costs consisted of indirect and direct medical costs as well as indirect non-medical costs. Volume data on inpatient and outpatient hospital care in the AMC were gathered for the full period February 2004 to November 2010 from the local hospital information system at patient level. Data on use of inpatient and outpatient health care, and sick leave were gathered quarterly by patient questionnaires between November 2008 and December 2010. All unit costs were estimated for the base year 2009. The costs, outside the AMC were calculated using the reference costs from the national health care database.

**Base case analysis**

In the base case analysis, the simulated cohort consists of males, females or both males (50%) and females (50%). In case a patient reaches the symptomatic state, ERT is started. Utilities for untreated and treated males and females were assumed to be similar. Similarly costs for untreated and treated males and females were the same, depending on the disease state, with the exception of the costs of ERT in case of initiation of ERT.

In daily practice, acroparesthesia can be an indication for ERT. However as there are no data published yet and the AMC data are too limited to evaluate the implication of starting relatively early in the course of disease, this scenario was not performed.

**Scenario analyses**

To evaluate certain treatment strategies and the impact of utilities, scenario analyses were conducted on the base case scenario. The following scenario analyses were performed for males and females:

(1) start of ERT at the age of 40 years. This is based on the fact that the mean age of start of ERT of the entire ERT cohort is 40.6 years and is comparable for males and females. It is expected that due to family screening and increased awareness the age of diagnosis will be earlier and patients will be referred to the AMC clinic at a younger age. As a consequence ERT may be initiated at a younger age and this is simulated in the base case where patients are treated as soon as symptoms are present.

(2) lower QoL during the natural course. The implications of a lower QoL in the untreated cohort was evaluated based on the literature. Data on change of health utility during the natural course and treated course are limited but it has been described that in a cohort of Fabry patients that initiated ERT the pre-treatment health utility increased from 0.64 to 0.74 [5]. The health utility scores during treatment were comparable to the AMC cohort (mean health utility 0.77). Based on these data an assumption was made that during the natural course, the utilities in each disease state were 0.1 (0.74-0.64) lower compared the utilities generated in case of ERT initiation.

(3) course of disease in patients with the classical phenotype only. Patients with an atypical phenotype have a more attenuated course of disease than patients with a classical phenotype [6]. The yearly probabilities were recalculated for the cohort without the patients with the atypical phenotype.

(4) the natural course of disease in case all patients were treated with ACE-inhibitors or angiotensin-receptor blockers. For Fabry disease there are no data published on the course of disease in patients treated with ACE-inhibitors or angiotensin-receptor blockers (ARB) as most of these have an indication and are treated with ERT. To estimate the possible impact of ACE and ARB medication, data from studies in large cohorts, at risk for renal and cardiovascular events were used to estimate the risk reduction of this type of medication[7-11]. For calculation of the risk reduction the most beneficial effect of ACE-ARB was modeled (hazard ratio of 0.70 during 2 years of follow-up) and applied to the symptomatic, and first complication group. The yearly risk reduction was calculated by correction with the Markov correction.

(5) in case patients with more advanced disease, i.e. the second complication group, would not be treated with ERT, as previous studies have doubted the beneficial effect of more severely affected patients.

(6) adding indirect costs.

**Sensitivity analyses**

The robustness of outcome was investigated by sensitivity analyses. This was performed through different procedures. A Monte Carlo simulation was performed to investigate the uncertainties of the yearly probabilities that were calculated. In this simulation, beta distributions were assumed for the yearly probabilities. As the AMC cohort and subgroups in this cohort were small, inherently due to the rarity of the disease, the number of patients in the actual cohort entering a new state could be less than one during one Markov cycle. In the analyzed cohort, it would be possible that no patients enter a next disease state in case the yearly probability is low. To be able to enter real integers in the Monte Carlo simulation (reflecting at least one patient experiencing an event), the lowest number of patients needed in the disease state to pass to the next state was calculated by dividing one by the yearly probability.

**3. Results**

**Yearly probabilities**

In total, data were available of 142 (58 males and 84 females) patients. At the first visit at the AMC none of them received ERT treatment.

*Developing acroparesthesia and other symptoms*

The age of the first presentation of acroparesthesia was estimated in current pediatric cohort (n=26). Of the 11 males, 50% had developed acroparesthesia at the age of 8 years old. The yearly probability was calculated by the proportion (0.50) divided by the median time (8 years). If corrected with the Markov factor the yearly probability of developing a first symptom was tp = 1 - (1 – 0.50)^1/8^ =0.083. Of the 15 females, 0.44 had developed acroparesthesia at the age of 12. The calculated yearly probability was 0.047.

To calculate the time to the presence of the first additional symptom (as defined previously) as accurately as possible, this was evaluated in the pediatric cohort and young adults that were referred through the pediatric outpatient clinic. This is a representative cohort, as this cohort represents both symptomatic and asymptomatic patients, mostly diagnosed through one of their adult family members. In total data of 36 patients fulfilled the criteria, of which 3 pediatric males and 3 pediatric females had no complete evaluation yet and were not included in the analysis. Of this cohort (12 males and 18 females), the median time the first symptoms was 17.4 years. The yearly probability was calculated by the proportion (0.50) divided by the median time to the first symptom (17.4 years). If corrected with the Markov factor the yearly probability of developing a first symptom was 0.039.

The rate of developing another symptom (as defined previously) was the same for the pediatric patients with and without acroparesthesia (probably due to the small size of the cohort). Therefore the 50% of the males and females with acroparesthesia would develop a symptom within 9.4 (17.4-8) and 5.4 (17.4-12) years respectively. Therefore the rate of developing a symptom in case of acroparesthesia in females was higher compared to males.

*Symptoms, developing the first complication: ESRD, a cardiac complication, CVA or death.*

At the time of the first evaluation at the AMC, 48 females presented with symptoms compared to 46 males. At that time 7 females had developed the first complication and the median time to the first complication was 66.0 years. Of the 46 males, 12 males had experienced a first complication after a median age of 53.3 years. The time patients developed their first symptom could not be ascertained for most (adult) patients. Some patients were diagnosed relatively late in the course of disease while probably already having undiagnosed symptoms for years. Therefore the median time to the first presentation of symptoms in the pediatric cohort was used. Therefore the median time between the first presenting symptoms and the first complication for males was (53.3-17.4) 35.9 years. The yearly probability was calculated as the proportion of patients that developed a cardiac complication, ESRD, stroke, or death, divided by 35.9 years, corrected with the Markov correction. In females, the time to the first complication was 66.0 years. Similarly to males, the median time to the first presenting complication was calculated as (66.0-17.4) 48.6 years.

For the estimation of the treatment effect, all data from the treated and untreated cohort of 100 symptomatic patients as of December 2010 were included in the analysis as described elsewhere (Rombach et al, manuscript included ). Gender, age and ERT duration all significantly contributed to the prediction of a first complication.

**Table 3. Multiple logistic regression analysis for the development of a first complication with gender, age and ERT duration as predictors in a group of symptomatic patients.** The odds ratio represents the additional risk of developing a complication per year increase of age and per each year of ERT treatment. Males have an increased (baseline) risk compared to females.

|  | Parameter estimate (SE) | Odds ratio (95% CI) | *p*-value |
| --- | --- | --- | --- |
| Intercept | -3.024 (0.997) | - | 0.002 |
| Age | 0.045 (0.018) | 1.05 (1.0-1.1) | 0.012 |
| Gender (male) | 1.494 (0.51) | 4.45 (1.6-12.1) | 0.003 |
| ERT duration in years | -0.212 (0.088) | 0.81 (0.68-0.96) | 0.015 |

For estimation of the relative risk reduction, the median treatment duration was used. The odds ratio for 4.2 years of ERT would be 0.41. Calculating the risk reduction by odds/(1+odds) for 4.2 years, corrected for the Markov correction, resulted in a relative risk reduction of 0.12 per year.

*A first complication, developing a second complication in a different end-organ*.

The time from the first to the second complication did not differ between males and females. Of the 19 patients that developed a first complication 5 had developed a second complication before treatment could have been initiated. It was estimated through the Kaplan –Meier curve that 42% had developed a complication after 11.3 years. This included all patients, both with typical and atypical phenotypes. In case only classical patients were selected 50% had developed a second complication after 11.3 years.

As previously described (Rombach et al, manuscript included) investigating the contribution of gender, age and ERT duration, only ERT duration appeared to contribute significantly to the prediction of a second complication. The odds ratios are shown in table 4. In case age and gender were left out of the analysis, the decrease of the odds for a second complication remained the same however the p-value was more robust (0.53 (95% CI: 0.34-0.85), *p*=0.008).

**Table 4. Multiple logistic regression analysis for the development of a second complication on gender, age and ERT duration as predictors in a group of patients with a previous complication.** The odds ratio represents the additional risk of developing a second complication per year increase of age (although not significant) and per each year of ERT treatment. There was no significant difference in the odds of a second complication between males and females.

|  | Parameter estimate (SE) | Odds ratio (95% CI) | *p*-value |
| --- | --- | --- | --- |
| Intercept | -0.326 (2.967) | - | 0.91 |
| Age | 0.015 (0.046) | 1.02 (0.93-1.1) | 0.75 |
| Gender (male) | 1.027 (0.995) | 2.8 (0.40-19.6) | 0.30 |
| ERT duration in years | -0.647 (0.264) | 0.52 (0.31-0.88) | 0.014 |

For estimation of the relative risk reduction, the median treatment duration was used. The odds ratio for 2.3 years of ERT would be 0.23. Calculating the risk reduction by odds/(1+odds) for 2.3 years, corrected for the Markov correction, resulted in a relative risk reduction of 0.35 per year.

*A second complication, developing the third complication.*

In the untreated cohort, only three patients were still alive and all developed a third complication within the first year of follow-up. As a consequence, the yearly probability was high.

Of the nine patients who were still alive after the second complication, four developed a fourth complication or died; two with and two without treatment. As reported elsewhere (Rombach et al, manuscript included) in case of a third complication none of the independent predictors, including age, gender or ERT duration contributed significantly (table 5). However the group with the second complication and still alive was small. In the model no risk reduction of ERT was modeled in the second complication state.

**Table 5. Multiple logistic regression analysis for the development of a third complication.** Due to the small group, only ERT duration as predictors was included.

The odds ratio represents the additional risk of developing a third complication could indicate a beneficial impact of ERT however the groups are too small to draw any definitive conclusions.

|  | Parameter estimate (SE) | Odds ratio (95% CI) | *p*-value |
| --- | --- | --- | --- |
| Intercept | 1.102 (1.175) | 3.012 | 0.35 |
| ERT duration in years | -0.326 (0.223) | 0.722 (0.466- 1.118) | 0.14 |

*A third complication, developing a fourth complication (death)*

There were too few data available of this group (n=1). The death rate was assumed to be similar as in the third complication group.

**Utility scores**

The utility scores were collected prospectively as described below.

*Patients*

Ninety-six patients participated in filling out the EQ5D questionnaire, 34 patients did not receive ERT treatment during the entire follow-up period, 60 received ERT treatment or did in the past and 2 patients started ERT the during follow-up period and filled out questionnaires during both the untreated and treated period. In the untreated group 4 disease states and in the group receiving ERT 7 different disease states were identified.

*The untreated group*

The untreated group consisted of 6 males and 30 females. The age at start of the first questionnaire was 41.8±13.3 (median 41.8, range 19.7-73.1) years. The median follow-time was 1.9 years (0.3-2.1) and the median number of questionnaires filled out during the follow-up period was 5.5 (range 1-8) per patient. One female, that developed symptoms, entered a new state. As a consequence there were data available of 37 disease state periods.

*The treated group*

One male patient in the symptomatic state, did not fill out the questionnaire completely which was discarded. At start of collection of EQ5D questionnaires, the group consisted of 29 males and 31 females that received ERT for a median follow-up of 4.3 (range 0.01-9.3) years. Age at the first questionnaire was 44.8±15.4 (median 47.2, range 18.6-75.8) years. The median follow-up was shorter than for the untreated group as data were partly collected during the shortage period and were left out of this analysis. The median follow-up was 1.1 (0.3-2.1) years. The median number of questionnaires filled out was 3.9 (1-8). In total there were EQ5D scores results of 60 patients with data of 62 disease state periods. During follow-up two males developed cardiac complications and progressed to the state of cardiac complications.

**4. Model validation**

For validation of the model data from the literature were used and evaluated by expert opinion.

*Disease states*

In the model there is always a fixed progression of disease states from asymptomatic, through a symptomatic state towards complication(s). We analyzed the validity of this hypothesis and found that indeed no patients progressed from the asymptomatic state to a complication state or developed multiple complications in different organ systems at the same time.

*Natural history data on developing symptoms*

There are several natural history studies reports, on the first presenting symptom, however mostly referring to the classical presenting symptoms, acroparesthesia, anhidrosis and angiokeratoma, cornea verticillata and abdominal pain, usually presenting during early childhood in both males and females [12-16]. These first symptoms present in general five years later in females as compared to males and before development of LVH, CKD or white matter lesions [12-18]. In the present study, the first symptoms were defined as, acroparesthesia, LVH, CKD and the presence of white matter lesions. As LVH, CKD and white matter lesions (addressed in the Format Farmacotherapeutic dossier) are considered early symptoms for development of end-organ complications, symptoms in this study were defined differently compared to most literature studies [18-21].

There are studies that reported on the development of LVH, CKD or white matter lesions, but most of these studies, reported on the development of these outcomes separately and were not combined, as in the Markov analysis. In addition most studies, describe pediatric or adult (or both pediatric and adult) cohorts and the proportion of patients that has developed a certain symptom. Of these patients, the mean age of first presentation of the symptom is described, however this does not provide information on the other subjects that have not yet developed the symptom. Therefore survival analyses are needed. Only few studies reported survival curves on symptoms as well and are discussed in more detail [19, 22, 23].

Survival curves from the natural history in a population of 40 males and 36 females, showed approximately 50% of males and females developed LVH at the age of 45 and 60 years respectively [22]. Furthermore, there are several reports on the first presentation of LVH in males. In a natural history cohort, LVH presented at the age mean age of 42 (95% CI of 37.9-45) [12]. A similar mean was reported in a registry cohort of 124 males, with a mean age of first LVH at the age of 42.0 years [23]. In the same study, the mean age of development was almost ten years later in 54 out of 254 females who developed LVH at the age of 50.1 years. A similar age was reported in 35 of 248 females with LVH, with a mean age of onset of 50.4 years [16]. Other studies reported on cardiovascular symptoms in which the age for both males and females are lower however these also included other cardiac symptoms, not always specifically reported [14, 15]

Renal symptoms (especially microalbuminuria/proteinuria or decreased GFR ) usually present earlier than cardiac symptoms, both in males and females [14]. One natural history report showed the survival curves for the development of proteinuria and reported that 50% of the males with a GFR>90 ml/min/1.73 m^2^, had developed proteinuria in their forties and females in their fifties [19]. Several studies reported on the first renal symptom, in most studies not further specified whether this means microalbuminurie, proteinuria and or decreased GFR [14, 15]. In a large registry study, 17% of 713 males presented with renal symptoms at a median age of 20 years, and 11% of 430 females at the median age of 28 years, most likely referring to microalbuminurie or proteinuria although this was not further specified [15]. In a cohort of 15 pediatric males with mean age of 12.4 (1-21), proteinuria was already present in 13% [24]. In the 20 females, with a mean age of 12.7 (range 1.5-20), 15% showed already proteinuria [24]. In another large cohort of 248 females, 23.8% of the females showed proteinuria at the mean age of 38.8±18.2 years.

There are few studies on the development and presence of white matter lesions and no literature studies on the development of white matter lesions (estimated with Kaplan Meier curves). In a study on 50 male patients, with 4 year of follow-up, 37.5% had white matter lesions on the MRI at the age of 43 years. In a cohort of 27 patients, 13 males with a mean age of 36.6 years, 6 (46%) patients showed white matter lesions, and 9 of 14 (64.2%) females with a mean age of 39.7 years [25] but the number of patients was small. In a retrospective MRI study in 43 Fabry patients, including 25 males, with a mean age of 41.9 years old and 18 females, with a mean age of 52.5 years old, MRI images were abnormal in 16/25 (64%) males and 13/16 (81.3%) females.

In conclusion, based on the literature, the age of the first manifestations of LVH is around 40 years in males that develop LVH and 50 years in females that develop LVH. Microalbuminuria and white matter lesions usually develop earlier during the course of disease. Furthermore some patients may even not develop some of these symptoms. However, based on the available literature it seems likely that males with a classical disease phenotype develop one or more of these symptoms before the age of 40 and in females around 50. This is most likely an overestimation because assessments can be delayed (delayed diagnosis, or the patient is not symptomatic) and the age of first presentation is probably earlier.

**Comparison to AMC data**: In the current Markov analysis, 99% of the males develop a symptom at the age of 35 years, and 99% of the females at the age of 50 years. This is well in line with the data from the literature (expert opinion).

*Developing complications: natural history data*

There are several publications on the presentation of the first complications in Fabry disease, although few on the cumulative incidence of complications and mortality rates [12, 13, 17, 18, 22, 26]. As shown in survival curves of studies with large cohorts, mainly registry studies, with age the event rates increase [17, 18]. At the age of sixty, 60% of males has developed any complication (renal, cardiac or cerebrovascular event), at the age of 90 years, the rate has increased to 100%. In females, the event rate at the age of 60 was around 25%, at the age of ninety this increased up to 80%. Another study, that combined renal, cardiac, stroke and death reported somewhat higher rates [18]. All males had developed a complication at the age of 60 years, in females 90% had developed a complication at the age 70 years. However in this study cardiac events were defined as any cardiac event, also including bradycardias. This is slightly different from the definition in present study. Bradycardia is frequent and approximately present in half of the patient population, sometimes as the only manifestation of Fabry disease (personal communication).

**Comparison to AMC data:** Kaplan-Meier curves show similar results compared to the AMC untreated cohort, as almost all patients (males and females) have developed a complication at the age of 70 years. In the Markov analysis, in males at the age of 70 years, none of the patients turned out asymptomatic, 40% was still in the symptomatic state and the remaining proportion had developed a cardiac, renal or cerebral event or died. In females, none of the females was still asymptomatic, 51% was in the symptomatic state and the remaining developed one or more complications of whom 33% died. For both males and females, this was more or less comparable to the complication rate in the Registry study [17] and lower as predicted by the other natural history study [18] (expert opinion).

*Distribution of ESRD, cardiac complications and CVA in the Markov analysis.*

**Comparison to AMC data:** In the Markov analysis, 13% of the male population alive at the age of 70 years, had cardiac complications; the probability that a patient experienced a CVA and was still alive was 5%. Less than 1% of the males was in the ESRD disease state, because of the low probability of developing ESRD based on the prevalence of ESRD in the AMC cohort.

For the females, at the age of 70, the probability of being alive and a history of CVA was 4%, for cardiac complications 9% and for ESRD less than 1 %. 33% died at that time.

There is a discrepancy between the AMC untreated cohort and the data found in the literature with regard to ESRD. The prevalence of ESRD in natural history studies is estimated to be 13-18% while this was in the Dutch cohort the 8.6 % which most likely reflects improvement in health care in preventing ESRD (expert opinion).

*Mortality*

Previous data on mortality from the Dutch historic cohort showed the median age of survival is 57 (95% CI: 52-62) years in males and 72 (95% CI 69-75) years in females, a somewhat higher life expectancy than previously published [12, 13].

**Comparison to AMC data:** In the Markov model, the probability that a male patient died is 0.25 as apposed to 0.50 at the age of 57 in the historic cohort. In females, the probability of death at the age of 70 is also lower to the compared previous reports (0.35 in stead of 0.50 at the age of 72 years).

As these patients consisted mainly of patients born at the beginning of the century, survival has most likely improved to some extent due to improved supportive care (including dialysis, kidney transplantation, improved cardiac procedures and additional medication) (expert opinion). In addition the natural history cohort consisted of a higher number of patients with an atypical phenotype, probably also contributing to a somewhat lower mortality rate (expert opinion). However, in the analysis only including classical Fabry disease patients, the mortality rate for males at the age of 70 increased only slightly from 41% up to 43%. In the base case scenario the median age of the first complication was estimated to be 53 years. Therefore males with a severe phenotype, presenting with their first complication in their forties, will have a higher risk of death compared to males presenting their first symptom at the median age of 53 years.

*ERT data on developing complications*

Few studies have been reported on clinical events during ERT [27-31]. These studies reported on less than 5 years of follow-up after start of ERT and did not evaluate the contribution of age, gender and ERT duration on the outcome. There is only one placebo-controlled trial published on clinical events during treatment with agalsidase beta however with a mean follow-up of 1.5 years [27]. The relative risk reduction in this cohort was estimated (hazard ratio of 0.47 (95% CI 0.21-1.03, p=0.06) during a median follow-up of 18.5 months). Calculating the yearly risk reduction and correcting with the Markov correction, resulted in a yearly risk reduction of 0.40. This was comparable to the risk reduction in the group of patients with one complication (estimated yearly risk reduction of 0.35)

In the life time Markov model the transition probability in the disease states with two or more complications is high due to the high mortality rate.

Reference List

1. Rodenburg - van Dieten HEM. **Richtlijnen voor farmaco-economisch onderzoek; evaluatie en actualisatie.** *Diemen: College voor zorgverzekeringen* 2005.

2. Drummond MF, Jefferson TO. **Guidelines for authors and peer reviewers of economic submissions to the BMJ. The BMJ Economic Evaluation Working Party.** *BMJ* 1996; **313**(7052):275-283.

3. Sculpher M, Fenwick E, Claxton K. **Assessing quality in decision analytic cost-effectiveness models. A suggested framework and example of application.** *Pharmacoeconomics* 2000; **17**(5):461-477.

4. Briggs A, Sculpher M. **An introduction to Markov modelling for economic evaluation.** *Pharmacoeconomics* 1998; **13**(4):397-409.

5. Hoffmann B, Garcia de LA, Mehta A, Beck M, Widmer U, Ricci R. **Effects of enzyme replacement therapy on pain and health related quality of life in patients with Fabry disease: data from FOS (Fabry Outcome Survey).** *J Med Genet* 2005; **42**(3):247-252.

6. Aerts JM, Groener JE, Kuiper S, Donker-Koopman WE, Strijland A, Ottenhoff R, et al. **Elevated globotriaosylsphingosine is a hallmark of Fabry disease.** *Proc Natl Acad Sci U S A* 2008; **105**(8):2812-2817.

7. Yusuf S, Sleight P, Pogue J, Bosch J, Davies R, Dagenais G. **Effects of an angiotensin-converting-enzyme inhibitor, ramipril, on cardiovascular events in high-risk patients. The Heart Outcomes Prevention Evaluation Study Investigators.** *N Engl J Med* 2000; **342**(3):145-153.

8. HOPE investigators. **Effects of ramipril on cardiovascular and microvascular outcomes in people with diabetes mellitus: results of the HOPE study and MICRO-HOPE substudy.** Heart Outcomes Prevention Evaluation Study Investigators. *Lancet* 2000; **355**(9200):253-259.

9. Dagenais GR, Yusuf S, Bourassa MG, Yi Q, Bosch J, Lonn EM, et al. **Effects of ramipril on coronary events in high-risk persons: results of the Heart Outcomes Prevention Evaluation Study.** *Circulation* 2001; **104**(5):522-526.

10. Schadlich PK, Brecht JG, Rangoonwala B, Huppertz E. **Cost effectiveness of ramipril in patients at high risk for cardiovascular events : economic evaluation of the HOPE (Heart Outcomes Prevention Evaluation) study for Germany from the Statutory Health Insurance perspective.** *Pharmacoeconomics* 2004; **22**(15):955-973.

11. Hackam DG, Duong-Hua ML, Mamdani M, Li P, Tobe SW, Spence JD, et al. **Angiotensin inhibition in renovascular disease: a population-based cohort study.** *Am Heart J* 2008; **156**(3):549-555.

12. MacDermot KD, Holmes A, Miners AH. **Anderson-Fabry disease: clinical manifestations and impact of disease in a cohort of 98 hemizygous males.** *J Med Genet* 2001; **38**(11):750-760.

13. MacDermot KD, Holmes A, Miners AH. **Anderson-Fabry disease: clinical manifestations and impact of disease in a cohort of 60 obligate carrier females.** *J Med Genet* 2001; **38**(11):769-775.

14. Mehta A, Ricci R, Widmer U, Dehout F, Garcia de LA, Kampmann C, et al. **Fabry disease defined: baseline clinical manifestations of 366 patients in the Fabry Outcome Survey.** *Eur J Clin Invest* 2004; **34**(3):236-242.

15. Eng CM, Fletcher J, Wilcox WR, Waldek S, Scott CR, Sillence DO, et al. **Fabry disease: baseline medical characteristics of a cohort of 1765 males and females in the Fabry Registry.** *J Inherit Metab Dis* 2007; **30**(2):184-192.

16. Deegan PB, Baehner AF, Barba Romero MA, Hughes DA, Kampmann C, Beck M. **Natural history of Fabry disease in females in the Fabry Outcome Survey.** *J Med Genet* 2006; **43**(4):347-352.

17. Wilcox WR, Oliveira JP, Hopkin RJ, Ortiz A, Banikazemi M, Feldt-Rasmussen U, et al. **Females with Fabry disease frequently have major organ involvement: lessons from the Fabry Registry.** *Mol Genet Metab* 2008; **93**(2):112-128.

18. Schiffmann R, Warnock DG, Banikazemi M, Bultas J, Linthorst GE, Packman S, et al. **Fabry disease: progression of nephropathy, and prevalence of cardiac and cerebrovascular events before enzyme replacement therapy.** *Nephrol Dial Transplant* 2009; **24**(7):2102-2111.

19. Wanner C, Oliveira JP, Ortiz A, Mauer M, Germain DP, Linthorst GE, et al. **Prognostic Indicators of Renal Disease Progression in Adults with Fabry Disease: Natural History Data from the Fabry Registry.** *Clin J Am Soc Nephrol* 2010.

20. Patel MR, Cecchi F, Cizmarik M, Kantola I, Linhart A, Nicholls K, et al. **Cardiovascular events in patients with fabry disease natural history data from the fabry registry.** *J Am Coll Cardiol* 2011; **57**(9):1093-1099.

21. Fellgiebel A, Keller I, Marin D, Muller MJ, Schermuly I, Yakushev I, et al. **Diagnostic utility of different MRI and MR angiography measures in Fabry disease.** *Neurology* 2009; **72**(1):63-68.

22. Kobayashi M, Ohashi T, Sakuma M, Ida H, Eto Y. **Clinical manifestations and natural history of Japanese heterozygous females with Fabry disease.** *J Inherit Metab Dis* 2008.

23. Linhart A, Kampmann C, Zamorano JL, Sunder-Plassmann G, Beck M, Mehta A, et al. **Cardiac manifestations of Anderson-Fabry disease: results from the international Fabry outcome survey.** *Eur Heart J* 2007; **28**(10):1228-1235.

24. Ries M, Ramaswami U, Parini R, Lindblad B, Whybra C, Willers I, et al. **The early clinical phenotype of Fabry disease: a study on 35 European children and adolescents.** *Eur J Pediatr* 2003; **162**(11):767-772.

25. Fellgiebel A, Muller MJ, Mazanek M, Baron K, Beck M, Stoeter P. **White matter lesion severity in male and female patients with Fabry disease.** *Neurology* 2005; **65**(4):600-602.

26. Vedder AC, Linthorst GE, van Breemen MJ, Groener JE, Bemelman FJ, Strijland A, et al. **The Dutch Fabry cohort: diversity of clinical manifestations and Gb3 levels.** *J Inherit Metab Dis* 2007; **30**(1):68-78.

27. Banikazemi M, Bultas J, Waldek S, Wilcox WR, Whitley CB, McDonald M, et al. **Agalsidase-beta therapy for advanced Fabry disease: a randomized trial.** *Ann Intern Med* 2007; **146**(2):77-86.

28. Schiffmann R, Ries M, Timmons M, Flaherty JT, Brady RO. **Long-term therapy with agalsidase alfa for Fabry disease: safety and effects on renal function in a home infusion setting.** *Nephrol Dial Transplant* 2006; **21**(2):345-354.

29. Breunig F, Weidemann F, Strotmann J, Knoll A, Wanner C. **Clinical benefit of enzyme replacement therapy in Fabry disease.** *Kidney Int* 2006; **69**(7):1216-1221.

30. Tahir H, Jackson LL, Warnock DG. **Antiproteinuric therapy and fabry nephropathy: sustained reduction of proteinuria in patients receiving enzyme replacement therapy with agalsidase-beta.** *J Am Soc Nephrol* 2007; **18**(9):2609-2617.

31. Benichou B, Goyal S, Sung C, Norfleet AM, O'Brien F. **A retrospective analysis of the potential impact of IgG antibodies to agalsidase beta on efficacy during enzyme replacement therapy for Fabry disease.** *Mol Genet Metab* 2009; **96**(1):4-12.
